# Contrasting Metabolic Insufficiency in Aging and Dementia

**DOI:** 10.14336/AD.2021.0104

**Published:** 2021-07-01

**Authors:** Dennis A Turner

**Affiliations:** ^1^Neurosurgery, Neurobiology, and Biomedical Engineering, Duke University Medical Center, Durham, NC 27710, USA.; ^2^Research and Surgery Services, Durham Veterans Affairs Medical Center, Durham, NC 27705, USA.

**Keywords:** brain metabolism, neurovascular coupling, stroke, mitochondria, glucose transport

## Abstract

Metabolic insufficiency and neuronal dysfunction occur in normal aging but is exaggerated in dementia and Alzheimer’s disease (AD). Metabolic insufficiency includes factors important for both substrate supply and utilization in the brain. Metabolic insufficiency occurs through a number of serial mechanisms, particularly changes in cerebrovascular supply through blood vessel abnormalities (ie, small and large vessel vasculopathy, stroke), alterations in neurovascular coupling providing dynamic blood flow supply in relation to neuronal demand, abnormalities in blood brain barrier including decreased glucose and amino acid transport, altered glymphatic flow in terms of substrate supply across the extracellular space to cells and drainage into CSF of metabolites, impaired transport into cells, and abnormal intracellular metabolism with more reliance on glycolysis and less on mitochondrial function. Recent studies have confirmed abnormal neurovascular coupling in a mouse model of AD in response to metabolic challenges, but the supply chain from the vascular system into neurons is disrupted much earlier in dementia than in equivalently aged individuals, contributing to the progressive neuronal degeneration and cognitive dysfunction associated with dementia. We discuss several metabolic treatment approaches, but these depend on characterizing patients as to who would benefit the most. Surrogate biomarkers of metabolism are being developed to include dynamic estimates of neuronal demand, sufficiency of neurovascular coupling, and glymphatic flow to supplement traditional static measurements. These surrogate biomarkers could be used to gauge efficacy of metabolic treatments in slowing down or modifying dementia time course.

There are multiple proposed underlying hypotheses of Alzheimer’s disease (AD) and related therapeutic strategies, in relation to normal aging [1, 2]. These include metabolic/mitochondrial dysfunction leading to metabolic insufficiency [3-5], vascular dysfunction and stroke [6, 7], amyloid plaques [8], phosphorylated tau [3], early tau phosphorylation under low glucose conditions [9-12],and recurrent herpes viral infection [13]. Most animal models of AD, based on human mutations of either amyloid precursor protein (APP) or presenilin-1 (PS1), show progression of amyloid plaques and abnormal behavior, similar in many ways to the human aspects of worsening from normal aging to mild cognitive impairment (MCI) to frank dementia [14]. But the CVN-AD model (APPSwDI ^+/+^/mNos2^-/-^) also demonstrates metabolic impairment, intraneuronal tauopathy and phosphorylated tau, changes in neurovascular coupling, and severe neuronal degeneration and cognitive abnormalities, a process similar to AD, due to lack of mouse inflammatory nitric oxide synthetase (mNOS2) [3, 15, 16]. Thus, the CVN-AD model is more representative of the human condition, but with a reproducible degeneration that translates directly across age (Fig. 3).

Critical advantages of animal models of AD include being able to analyze any stage of the stereotypic disease progression (based on animal age), performing aging and gender analysis, and testing results of pre-symptomatic treatment to reveal critical aspects of metabolic derangements and mechanisms. In spite of model availability for testing therapeutic approaches, few approved treatments for AD are available (anticholinergic drugs, memantine), hence fresh approaches are critical [2, 4, 17-19]. Our hypothesis is that reduced brain glucose availability significantly contributes to neurodegeneration from a critical mismatch of dynamic metabolic supply and demand at an early point of degeneration, particularly in relation to age-matched controls. (schematically shown in Fig. 1).

CNS metabolism dynamically fluctuates in response to neuronal demand and metabolic need [20, 21] as well as circadian rhythm [22-24]. Neurovascular coupling is a key mechanism to transiently enhance blood flow and substrate delivery (ie, oxygen and glucose) to the brain to match neuronal demand [25]. A consistent finding early in AD is reduced glucose uptake into cells, typically measured with 18fluoro-deoxy-glucose (FDG) [26]. This significant reduction in glucose uptake could be due to: 1) abnormal neurovascular coupling with deficient substrate delivery [27, 28]; 2) microstructural aberrations of the blood-brain barrier (BBB) typical of AD [29]; decreased facilitated glucose transport across the blood brain barrier into the extracellular space [30]; and/or reduced metabolism within cells [26]. Vascular changes are also mirrored in a high incidence of stroke occurrence in Alzheimer’s, further impeding the critical flow of substrate into the brain [6]. Altered neurovascular coupling occurs in mouse Alzheimer’s models and in patients with both mild cognitive impairment [MCI] and overt AD [16, 31-36]. Many of these changes also occur with aging but are exaggerated in dementia syndromes [20].

This review discusses first the concept of metabolic insufficiency (1.), Alzheimer’s animal models and results (2.), metabolic treatment and biomarkers (3.) and a summary (4.).

**Figure 1.**
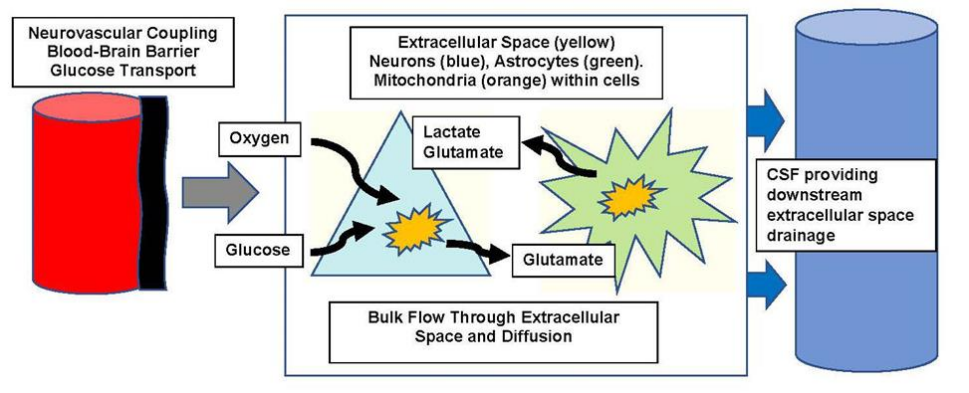
**This diagram shows the flow of substrate from the systemic vasculature (to the left), across the blood-brain-barrier to the extracellular space**. The flow is modulated by dynamic neurovascular coupling, which integrates neuronal demand into enhanced blood flow temporarily as needed. Glucose transporters are needed for glucose entry into the brain, but oxygen can diffuse once released from hemoglobin. The extracellular space allows diffusion of substrates into the brain and near cells while also allowing toxins and metabolites to diffuse downhill into CSF, termed glymphatic flow.

## 1.1 Concepts of Metabolic Insufficiency

**Figure 2.**
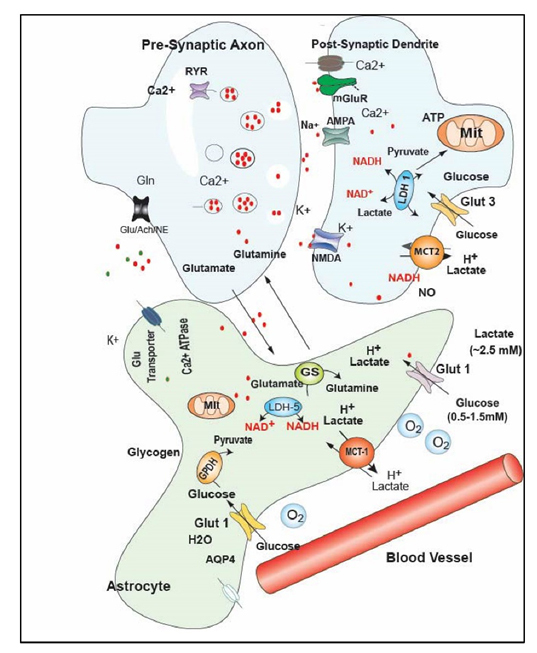
**This more detailed metabolic diagram is adapted from Shetty et al (Figure 3)** [21]. The diagram shows a blood flow with glucose transport and O2 diffusion into the extracellular space surrounding an astrocyte (green) and pre-synaptic and post-synaptic neuronal subparts (blue). In the astrocyte glucose is taken up by glucose transporter 1 (Glut1) and lactate extruded through monocarboxylate transporter 1 (MCT-1). Also shown in the astrocyte are aquaporin transporters (AQP4), glutamine synthase (GS), glycogen stores, lactate dehydrogenase (LDH-5), and glucose metabolism to pyruvate via glycerol phosphate dehydrogenase (GPDH). Mitochondria (MIT) also generate NADH from NAD+. In neurons Ca2+ (shown as red dots) is sequestered in endoplasmic reticulu (ER), glutamine is taken up and extruded through vesicles as glutamate. On the post-synaptic side glutamate binds to metabotropic glutamate receptors (mGluR) as well as AMPA and NMDA receptors. Neurons transport ketones and lactate through MCT-2 transporters and glucose through Glut 3 transporters. Lactate levels vary from ~ 1-2.5 mM and glucose 0.5-1.5 mM and both vary dynamically with neuronal activity.

Low glucose metabolism occurs pervasively in early AD [26, 37]. Possible factors include altered blood vessels and stroke [6], insufficient neurovascular coupling and/or inadequate glucose delivery to capillaries [7, 28], reduced glucose transport across the blood brain barrier [38], as well as intrinsic changes in metabolism within neurons, such as changes in oxidative phosphorylation [20, 39] and mechanistic target of rapamycin (mTor) and other regulatory alterations [40-42]. There may be also reduced bulk transport within extracellular space as well as diminished uptake from the extracellular space into cells [43], and intrinsic metabolic changes in cytoplasm and mitochondria decreasing energetic capability, particularly in neurons with aging [20, 44]. Bypassing glucose uptake, ketone bodies may provide alternative brain fuel as the disease progresses [14, 45, 46]. CNS metabolism is highly dynamic in response to rapidly changing neuronal demand and induced metabolic stress [47]. Therefore, real-time measurements of changes in substrate supply during increased metabolic demand are critical to unmask transient deficiency and cerebral metabolic rates of O2 and glucose. Static substrate levels in CSF or brain tissue [48] give only a time-averaged overview of metabolic pathways. Even short periods of inadequate substrate supply during high demand may lead to hypoglycemia or hypoxia and insufficient energetic capability, accelerating AD changes [21, 30, 49, 50].Covert hyperexcitability, excessive neuronal activity, and reduced metabolism for glutamate uptake may also contribute to a greater metabolic need that may be unmatched by supply [51], as well as enhanced glutamate and excitotoxicity [45, 52]. Many of these factors are shown in more detail in Fig. 2, which highlights more details of substrate movement from blood vessels into cells, many of which change in AD and aging [21].

## 1.2 Vascular Factors and Stroke

Stroke occurrence, abnormal metabolism, and loss of blood vessel regulation in the brain can together lead to neurodegeneration, the underlying mechanism of dementia syndrome [6, 53]. Stroke may be preventable through control of medical risk factors and systemic conditions. These risk factors include lipids, diabetes [54, 55], high blood pressure and ApoE status [56], which can all bias and worsen outcome. Though vascular dementia is considered separate from spontaneous AD [57] there are many abnormal cerebrovascular manifestations in AD patients, including lacunes, small vessel strokes and hemorrhages. Further, intrinsic factors such as tissue plasminogen activator (tPA) may also dysfunction with dementia syndrome and the presence of amyloid plaques [58]. Thus, dementia may be attributable to a number of vascular and cellular factors, synergistically leading to neuronal degeneration.

## 1.3 Neurovascular Coupling

Because neurovascular coupling is a dynamic process, measuring baseline blood flow during periods of low metabolism may not reveal true, underlying deficits. Hence, cerebral blood flow and substrate uptake need to be assessed during brain activation conditions where demand is high, to assess if supply can match this demand. Maximal brain activation occurs with high potassium levels around neurons in the brain [59] which requires significant metabolic support to restore neuronal homeostasis. Maximal neuronal activation with addition of potassium directly to the cortex causes a severe neuronal depolarization that spreads across the brain, termed spreading depression [59]. These spreading depression events do occur in humans but are triggered primarily after stroke and head injury where intrinsic damage causes high potassium levels [60].

However, abnormal metabolism and low glucose uptake are present even early in mild cognitive impairment, a transition in time and disease progression between normal aging and clear Alzheimer’s dementia [14, 61]. Brain metabolism interacts with blood vessel regulation through neuronal activity signaling, which functions to increase brain blood flow to accommodate the brain’s need for metabolic supply [27]. This dynamic regulation of blood flow by neuronal activity is called neurovascular coupling, where neurons, astrocytes and blood vessels interact to continuously match substrate supply (through blood flow) with ongoing metabolic requirements. In the transition between aging, mild cognitive impairment, and dementia both neurovascular coupling and blood brain barrier function are progressively perturbed [16]. These changes reduce supply of glucose to the brain, compounding the intrinsic reduction in the brain’s ability to metabolize glucose [7].

## 1.4 Blood-Brain Barrier Changes and Glucose Transport

Whereas oxygen can diffuse from hemoglobin in blood vessels across the blood brain barrier into the brain, glucose transport across the blood brain barrier must be facilitated by transport (glucose, GLUT1) carriers which are severely constrained in dementia [30]. Blunted neurovascular coupling together with diminished glucose transport can significantly reduce glucose entry into the brain [28]. If brain blood flow and/or glucose transport are truncated, then intermittent low glucose conditions can disrupt metabolism during heightened metabolic demand. These conditions may starve neurons, exaggerate neuronal damage, and worsen dementia [54]. As predicted from human glucose uptake studies showing that impaired metabolism may occur early in dementia [4, 26, 61], then the metabolic insufficiency may be present over years causing progressive degeneration and the severe atrophy and neuronal loss associated with dementia. If inadequate neurovascular coupling early in the disease could be estimated by surrogate biomarkers, such as metabolic brain studies with dynamic activation [62] then these patients may be more amenable to early metabolic treatment paradigms [61]. By acting early in the disease, the time course may be altered significantly.

## 1.5 Brain Parenchyma CSF and Glymphatic Flow

Extracellular space fluid arises from perivascular spaces, leakage across the blood brain barrier, and from cells, but eventually drains into CSF (Fig. 1), with lactate as a critical signal [63, 64]. The extracellular space volume is highly dynamic and represents ~ 15-20% of brain volume, varying with neuronal activity, sleep, and circadian rhythm [65-67]. In AD this drainage may be compromised resulting in abnormal protein clearance and plaques [43, 68, 69], which exacerbates stasis. Likewise, spreading depression decreases extracellular drainage due to cell swelling [65, 66] but subsequently increases vascular permeability with MMP-9 induction [70, 71]. Extracellular solute and substrate transfer are critical links between blood vessels and cells (Fig.’s 1, 2). Thus, measurement of extracellular metabolic intermediates (ie, glucose, glutamate, lactate, O2) could provide indirect analysis of abnormal substrate transfer and clearance pathways in this model of AD [68], separate from flow studies. Sluggish extracellular clearance into CSF may also result in accumulation of amyloid and plaque contents.

## 1.6 Intrinsic Cellular and Mitochondrial Metabolic Changes.

Cellular metabolic alterations may be difficult to separate out from vascular supply, neurovascular coupling, glucose transporter efficacy, and extracellular availability of substrate. One physiological approach to discriminate vascular and substrate supply factors is to prepare either acute brain slices from animals at various ages and stages of dementia development or isolated mitochondria, both similar to a biochemical biopsy at that point in time [72-76]. Brain slices are maintained in a metabolically stable environment with artificial CSF, glucose and oxygen supply, though do demonstrate some substrate gradients in the interior [51, 77, 78]. Isolated mitochondria can also be studied in an artificial environment but require substrate to be activated, but have shown complex I deficiency with aging, for example [79, 80]. But, the applied metabolic conditions in these in vitro preparations are consistent across age, genotype and other cofactors, to be able to define intrinsic, cellular metabolic changes separate from vascular substrate supply (Fig. 2).

Using this in vitro approach aging leads to decreased oxidative metabolism [20, 39], with more reliance on aerobic glycolysis and reduced resilience during anoxia and/or low glucose conditions [39]. Energetic capacity is also reduced in AD, even prior to amyloid plaque deposition [26, 81] - low extracellular substrate supply available to cells may starve mitochondria for substrate [54]. Our laboratory has had an extensive experience in analyzing cerebral metabolism and substrate supply during a wide variety of metabolic challenges in vitro and in vivo, including train stimulation, hyperexcitability [51], spreading depression [SD] and hypoxia-ischemia [20, 21, 39, 51, 73, 77, 78, 82-84]. These studies consistently show a significant shift in internal, cellular metabolism towards glycolysis and with reduced efficiency of mitochondrial function during aging[79, 85, 86].

## 2.1 Alzheimer’s models and Studies of Metabolic Insufficiency

**Figure 3.**
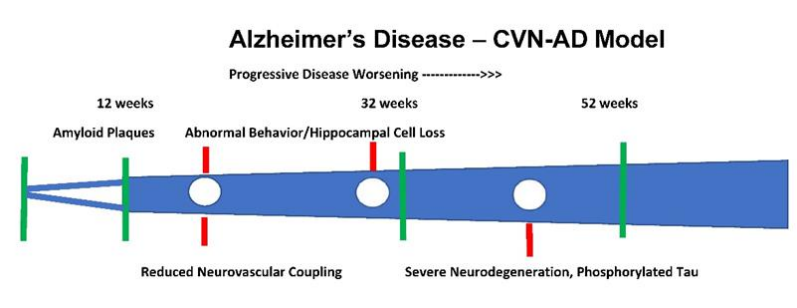
**Progression in CVN-AD Alzheimer’s model**. This diagram shows various phases of degeneration in this mouse model, with a short asymptomatic period prior to 12 weeks. By 12 weeks there is presence of amyloid plaques around blood vessels, abnormal behavior and hippocampal cells loss over 24-36 weeks [15, 87, 90]. There is abnormal neurovascular coupling by 24-32 weeks [16]. By 52 weeks the mouse model shows severe neurodegeneration, phosphorylated tau. This model shows severe, premature aging changes compared to age matched controls.

Neurovascular coupling and substrate supply were recently analyzed in neocortex in the CVN-AD Alzheimer’s mouse model[16, 87-91]. This CVN-AD model (APPSwDI +/+/mNos2-/-) of AD replicates extensively the histological and behavioral characteristics of the human disease including early onset of amyloid plaques by 12 weeks, phosphorylated tau by 24-36 weeks, and significant cell loss and behavioral changes by 36 weeks, replicating most human changes in pre-clinical and clinical aspects of AD (Fig. 3 shows the time course). The hippocampus is involved earlier and is more susceptible to early pathological changes than the neocortex, which we have extensively studied in our preliminary data [87, 88, 90]. Figures 1 and 2 show the metabolic flow of substrate delivery and utilization, from vessels to extracellular space, uptake into cells and mitochondria [21]. The flow includes glucose and O2 supply via neurovascular coupling and transport into extracellular space, cells, aerobic glycolysis, and mitochondrial metabolism. Extracellular flow includes a mixture of CSF influx from perivascular spaces, extracellular fluid transport across vessels, and secondary leakage from cells, all coursing downhill into CSF ventricles as a drain of extracellular solutes and toxins ([63, 92].

In animal models of AD neurovascular, cognitive changes and progressive dementia occur but in a compressed time course compared to the human transition between normal aging, mild cognitive impairment and dementia, over a stereotypic time course as a function of mouse age [87]. As in humans, age is a critical risk factor for development of dementia, including histological changes (ie, loss of neurons, development of plaques and tangles), behavioral abnormalities (ie, memory loss), and blood brain barrier changes. Animal age in dementia models directly translates into severity of degeneration and is more predictable than in humans. In animal models we can also induce spreading depression events as a function of age by adding a focal potassium source onto the brain, to more adequately test enhanced blood flow (and indirectly substrate) supply to the brain in response to a severe metabolic demand. By testing these spreading depression events across age we can compare spontaneous aging neurovascular responses occurring in control animals to responses affected by degeneration in the transgenic animal model with Alzheimer’s like stigmata [16, 93]. During spreading depression events there is also significant calcium increase within neurons, which may be intrinsically damaging through loss of control of calcium-mediated signaling [1]. Thus, the neurovascular and metabolic responses to spreading depression events and anoxia can help understand altered mechanisms of neurovascular coupling in dementia development. Additionally, these metabolic demands stringently test the reserve which the brain’s vascular supply demonstrates under metabolic stress, as a function of normal aging and dementia.

## 2.2 CVN Alzheimer’s Mouse Model and NOS Isoforms

The CVN-AD mouse model (APPSwDI +/+/mNos2-/-) is based on transgenic insertion into mice of a familial human APP mutation, but this mutation alone primarily leads to amyloid deposition. In pioneering studies Dr. Colton showed that high background levels of nitric oxide [NO] in mouse brain are neuroprotective compared to humans [15], due to enhanced mouse NOS2 (inflammatory nitric oxide synthetase) generation of NO. Her lab then deleted mouse NOS2 [15, 87, 90], leading to severe neurodegeneration, accelerated amyloid deposition (by 12-24 weeks; Fig. 2) and phosphorylated tau with tauopathy, the critical elements defining a “complete” Alzheimer’s model [15, 90]. The CVN-AD model recapitulates human Alzheimer’s stigmata but preserves transient, physiological NO signaling (for neurovascular coupling) through the two isoforms of NOS1 and NOS3. Appropriate controls for the CVN-AD model include the C57Bl background, mNOS2 -/-, and APPSwDI +/+ mice.

NOS2 is one of three isoforms of NOS: 1) NOS1, neuronal NOS [nNOS], is transiently generated by neurons as a critical signal for synaptic plasticity and neurovascular coupling [94]; 2) NOS2 [iNOS] is slowly generated by inflammatory cells (ie, microglia) and provides background levels as a signal to initiate inflammation; and 3) NOS3 or endothelial NOS [eNOS] is important for flow- and pressure related dynamic vascular regulation. Both nNOS and eNOS levels showed minimal changes with the congenital loss of NOS2 [15, 90]. Importantly, the critical neuronal functions which nNOS subserves (ie, neurovascular coupling) are intact in the CVN-AD model. We have confirmed the preservation of these important NO-signaling cascades and neurovascular coupling in the young CVN-AD and mNOS2 -/- animals [16, 28, 95].

## 2.3 Analysis of Neurovascular Coupling

We assessed systemic supply, neurovascular coupling, extracellular measurements of multiple substrates, and estimating neuronal demand across varying severity of demand, to define substrate supply limitations as a function of dynamic metabolism in the CVN-AD model in vivo. These physiological experiments are highly novel, assessing the dynamic nature of substrate supply and neuronal demand on a second by second basis with progressively more severe conditions to elucidate metabolic insufficiency and whether neurovascular coupling or glucose uptake (ie, diabetes type 3) are limiting features of the substrate cellular supply [96]. These dynamic metabolic demand experiments extended beyond baseline and resting conditions, where supply may be balanced with demand, to assess whether there is sufficient metabolically - induced demand to force a transient, supply/demand mismatch [84]. The overall glucose hypometabolism in AD may not simply be reduced supply but also changes in demand at the cellular level, which can be estimated from cerebral metabolic rates of O2 and glucose [37].

Importantly, the onset and progression of reduced neurovascular coupling and efficacy of metabolic supply in the CVN-AD model and AD may precede or aggravate histological amyloid changes expected at each age. However, as in humans, the Alzheimer’s model progresses on a background of “normal” aging, hence the critical comparison to the WT and genotype control animals with age, which also demonstrate progressive alterations in metabolism, neurovascular coupling and substrate supply in older mice. Many of these approaches are translatable to humans with imaging techniques to estimate metabolism on a real-time basis [28, 32, 97]. The overall finding of these studies was that CVN-AD results showed premature aging effects compared to the age matched controls [16].

These novel results show a significant reduction in neurovascular coupling in response to robust metabolic demand in middle-aged and aged CVN-AD mice compared to age-matched controls, confirming that a significant aspect of metabolic deficiency in AD may arise from insufficient, dynamic substrate delivery to the brain [28]. These reduced neurovascular responses may create a net deficit in substrate availability (ie, glucose and O2) to the cortex during maximal metabolic responses and likely exacerbate ischemia [28], particularly as the Alzheimer’s-like phenotype progresses [87, 98]. These blood flow results are parallel to pathological vascular changes occurring in neocortex in the middle-aged and aged CVN-AD mice, similar to amyloid angiopathy [98-100]. The comparison between CVN-AD animals and age-matched controls is critical, allowing separation of aging effects from AD-like pathological alterations.

## 2.4 Intrinsic Energetic Alterations

With aging we have found reduced oxidative capability and enhanced dependence on glycolysis using a brain slice approach [20, 39]. These mirror changes in mitochondrial complex I found with aging [79, 80]. Further research is planned to compare direct, cellular metabolic changes across the lifespan in the CVN-AD model, to complement alterations in neurovascular coupling and supply. Energetics and respirometry to measure the relative balance of glycolysis and oxidative phosphorylation may also be performed in brain slices as well as isolated mitochondria [101, 102].

**Table 1:** Metabolic Approaches to Alzheimer’s Disease.

Mechanistic Approach	Human Trials?	References
**BBB and vascular glucose uptake (Glut-1)**		[17]
GLP-1 agonist (glucagon-like peptide), liraglutide	yes	[129]
Metformin	yes	[145]
Diabetic and insulin treatment	yes	[55]
Ghrelin agonists (MK-0677)	yes	[146, 147]
**Extracellular space and clearance**		
enhancing extracellular fluid, clearance	no	[148]
**Aerobic glycolysis and neuronal glucose uptake (Glut-3)**		[149]
2-Deoxyglucose (antagonist to glucose)	no	
**Alternative mitochondrial substrates**		[150]
Ketones (ie, ketogenic diet, fasting)	yes	[14]
Caprylidene (precursor to ketone bodies)	yes	[46]
Oxaloacetate (mitochondrial precursor)	yes	[151]
**Mitochondrial functioning**		
NAD analogues/supplementation - nicotinamide	yes	[130]
Oxidative stress and antioxidants - Vit E + Vit C	yes	[130]
Thiamine deficiency - many early trials	yes	[44, 152]
Carbonic Anhydrase - acetazolamide	no	[153]
**Hyperexcitability, increased demand**		
Anticonvulsants, alternative diets	yes	[128]
**Neurovascular coupling (diminished vessel response)**		[28, 35]
losartan (Angiotensin II receptor)	yes	[154,155]
cholinergic/norepinephrine vascular tone	yes	[154]
**Metabolic regulatory dysfunction**		
mTor - rapamycin	no	[40-42]
Sirtuins and NADH regulation	no	[157]
Circadian rhythm dysfunction	no	[22, 23]

## 3.1 Biomarkers and Therapeutic Strategies for Metabolic Insufficiency

The understanding of metabolic insufficiency in AD will first require defining substrate availability and limitations to cells from the extracellular space, as detailed in Figure 1 (substrate flow diagram) and Table 1 (a summary of possible metabolic treatment schemes in both aging and AD). These are specific areas of treatment development from amongst a wider range of therapeutic opportunities [17]. Metabolic approaches include systemic supply of glucose and oxygen as critical substrates, neurovascular coupling, changes in the capacity for nutrient transport across the pathological vessel wall and blood brain barrier in AD, diffusion within the brain extracellular space, and availability of substrate at cell surfaces. After analyzing the extracellular availability of these substrates together with their integrated utilization by cells within the brain, the balance determines the available levels amongst several metabolic demands. The next step is to intervene, particularly in glucose availability, where metabolic insufficiency has been clearly determined in humans in both MCI and in AD [54, 61].

## 3.2 Biomarkers

There is a critical need to differentiate the stages between normal aging and dementia syndrome, particularly the early onset of mild cognitive impairment (MCI), so that early treatment could applied most effectively to the appropriate candidates. For this reason, a number of biomarkers have been tested, including imaging studies, cerebrospinal fluid samples (CSF), and more recently blood tests that may be easier to perform. Imaging studies, for example, include glucose metabolism (measured by PET 18fluoroglucose uptake into the brain) [61, 103, 104], structural imaging studies showing atrophy including the fornix [105-108], functional MRI and resting state MRI to characterize blood flow changes and network changes [109-111], and assessment of CSF clearance to estimate glymphatic flow [43, 63, 69, 112-114]. There are also studies prospectively analyzing the use of direct amyloid imaging to compare aging controls with early Alzheimer’s patients [115]. Using these image approaches neurovascular coupling can also be estimated through vascular reactivity, for example [110, 111, 116, 117].

CSF biomarkers have focused on lactate, amyloid, and tau, which filter down through the extracellular space and drain into CSF as a filtering system [61, 104, 118, 119]. Tau may show progressive phases with different subsets of the molecular appearing over time [11]. However, CSF requires a lumbar puncture and measures only static changes as metabolites accumulate in CSF slowly. Therefore, serum or blood markers may be much easier to obtain but also the metabolites are at much lower levels. In spite of these difficulties, several novel approaches for estimating blood biomarkers are currently being tested. These include analysis of peptides and metabolites, for example [120, 121]. Tau may also be detected in serum [10, 12, 122]. The relative efficacy of several of these biomarkers has also been assessed for early detection of AD [61, 103, 115, 122, 123].

Direct, dynamic recordings of brain glucose and oxygen under a variety of metabolic activation paradigms may reveal the underlying mechanisms of metabolic deficiency in comparison to the degeneration noted as a function of animal age [39, 78, 124]. Both substrates could be clinically measured with PET dynamic activation as a potential method to highlight patients for specific metabolic interventions [62]. Further, neurovascular coupling to a variety of activation paradigms may be tested in mild cognitive impairment and Alzheimer’s patients to understand the translation of these concepts to the clinical condition [125]. These could be tested with clinical activation including sensory or direct nerve stimulation. As in metabolic clinical studies (with FDG PET) comparing efficacy of resting imaging biomarkers to CSF studies [61] then additional dynamic biomarkers may be derived from maximal increases in blood flow in response to an external stimulus, for example. Further areas within the brain should also be explored with dynamic imaging responses to metabolic challenges, to understand region-specific susceptibility to degeneration, particularly hippocampus. Extension to human analysis of neurovascular coupling will also be critical, as well as studying treatment options focused on mechanisms of neurovascular coupling and rate-limiting steps of glucose uptake and utilization within the brain.

As these biomarkers are tested the concept of early, pre-clinical AD may be enhanced and confirmed, allowing earlier, pre-symptomatic treatment in which treatment schemes may show improved efficacy, as compared to more severe Alzheimer’s states [2, 6, 126].

## 3.3 Translational Therapeutic Opportunities

Critical deficiencies in metabolism are likely to include reduced neurovascular coupling, which can lead to insufficient metabolite being delivered at the capillary level to the brain, as well as decreased substrate supply [37]. There are many risk factors associated with AD, particularly APOE4 [127], but treatment strategies to improve risk factors are highly limited. For example, strategies proposed at a number of possible approaches are included in Table 1 [17]. Novel translational and clinical approaches to treat AD range from reducing demand due to ongoing (often undetected) hyperexcitability [52] (ie, anticonvulsants - [128]), improving neurovascular coupling with losartan (an Angiotensin II antagonist - [28]), to improving glucose transport across the blood brain barrier (liraglutide - GLP-1 agonist - [129]). Separately, there are alternative substrates bypassing the GLUT1 receptors, particularly various ketone bodies, oxaloacetate, and their precursors (ie, Caprylidene - [46]). Multiple mitochondrial approaches exist to treat known oxidative phos-phorylation changes, including nicotinamide supplementation to improve redox states and ATP production [83, 130], antioxidants, thiamine supplementation, and acetazolamide to enhance metabolism and reduce ROS [81]. There are two different approaches to studying mechanisms in mice: 1) acute experiments to detail specific mechanisms, points of action and dose-response functions; and 2) chronic experiments focusing on indirect effects, such as behavioral outcome and changes in histology [131]. The latter empirical approach does not reveal mechanisms of action, since full chronic dose response curves and drug availability need to be calculated and compared, and the outcome indices are indirect and vary with the Alzheimer’s model used.

## 3.4 Enhancing Glucose Availability

Even though reduced glucose metabolism has been consistently noted in clinical studies, treatments to address this issue remain empirical, though some are already in initial (but mixed outcome) clinical trials [129, 132]. One approach has been to enhance glucose transport across the blood brain barrier, mediated by Glut1 glucose transporters, through upregulation or enhancement of GLUT1 function [30]. Animal models have shown exacerbation in Alzheimer’s models with even partial antagonism of GLUT1, indicating its critical role in glucose transport and degeneration [30, 50], with reduced GLUT1 noted consistently in AD, as a partial approach to understanding reduce metabolism [133-136]. The primary approach to enhance GLUT1 function is through glucagon-like protein 1 agonists (particularly liraglutide), which have already been approved as a second line treatment for diabetes, though whether AD can be considered as a “diabetes type 3” remains controversial [96].

Key residual questions include the mechanisms underlying the defective neurovascular coupling, and whether even earlier changes occur in hippocampus, which shows a more progressive degeneration process than the cortical surface of the brain. If glucose transport is a critical limitation, this may be upregulated through glucagon-like-peptide (GLP-1) agonists to possibly prevent degeneration if caught earlier [38]. For example, clinical trials of GLP-1 agonists are proceeding in treating or preventing dementia syndrome in patients. If dynamic PET activation to sensory stimuli was able to differentiate patients early on [61], for example, this type of metabolic approach could be followed by such a biomarker for improvement, lending a personalized approach to therapy [62]. Mediators of neurovascular coupling may also be analyzed for an altered role, such as nitric oxide [94, 137-139]. Because the metabolic deficiency is primarily in glucose metabolism, ketone substitution as an alternative brain fuel has been predicted from clinical studies [14] and also applied in animal models of dementia [45]. These translational examples highlight how glucose metabolism and neurovascular coupling may be analyzed in a time/age/degeneration profile as well as regional brain assessment and rational interventions developed to improve metabolic supply.

## 3.5 Pre-Symptomatic Treatment

More detailed understanding of time-related progression of the dementia in relation to neurovascular coupling and substrate supply are critical diagnostic aspects. Using surrogate markers to estimate neurovascular coupling (in addition to baseline metabolism at rest) may help understand another dimension of time related progression from normal aging to mild cognitive impairment [62]. These surrogate markers could be followed with metabolic treatment, for example, to see if degeneration could be slowed, rather than wait for years for full AD symptoms to develop. Because age in dementia animal models provides an expected, surrogate measure of degeneration, this timeline is similar to the human (but much slower) progression from normal aging to minimal cognitive impairment to dementia. However, in the human progression these steps are much less clear and less well defined. Also, in animal models reliable early treatments prior to degeneration can be tested, similar to planned studies of earlier, less symptomatic patients [140-142]. However, the earlier the possible prediction of eventual dementia syndrome, the less accurate the prediction. If dementia diagnosis could be more securely established prior to severe degeneration occurring then more aggressive or invasive disease modifying treatments could be ethically applied at an early time point [61, 118, 120, 122, 143]. For example, in early Parkinson’s disease, L-dopa can be used as a diagnostic test to confirm the diagnosis, which has led to the concept of using, early invasive, deep brain stimulation as a potential disease modifying treatment compared to optimal medical therapy [144].

## 4. Conclusions

There is a cascade of neurovascular, glucose transport, and cellular metabolic changes early in AD progression, limiting substrate availability and utilization. But, specific mechanisms are needed to correlate the dynamic metabolic cascade with neuropathological worsening, particularly identifying rate-limiting steps. Understanding physiological metabolic demand, extracellular space, and pathological limitations leading to degeneration are optimally explored through physiological substrate demand/supply experiments at early stages of the Alzheimer’s-like progression. The dynamic nature of neurovascular coupling, substrate flow (ie, on a second-by-second basis) may also influence whether there is sufficient immediate metabolism for neuronal function. Progression, aging, and gender factors are critical to understand mechanisms of substrate availability and metabolic sufficiency.

In summary, the significant changes in neurovascular coupling that we report in the CVN-AD model of dementia show both early age onset compared to control animals and involvement of the neocortex, with regional susceptibility of the hippocampus to be more completely evaluated in later studies. These results may be translated using dynamic measurements of neurovascular coupling to provide additional time and region- based biomarkers in predicting early onset of mild cognitive impairment and degeneration in humans. There are several potential avenues of translational, metabolic therapy which could be applied early, such as enhanced glucose transport and ketone availability. Further, depending on the security of early diagnosis, more aggressive, invasive treatments may be tested, in parallel to another degenerative condition, Parkinson’s disease.
